# Individualizing the use of [^18^F]FDG-PET/CT in patients with complicated *Staphylococcus aureus* bacteremia: experiences from a tertiary care center

**DOI:** 10.1007/s15010-021-01740-4

**Published:** 2021-12-20

**Authors:** Eline J. van Leerdam, Michelle Gompelman, Renée A. M. Tuinte, Erik H. J. G. Aarntzen, Marvin A. H. Berrevoets, Ianthe Maat, Chantal P. Bleeker-Rovers, Reinout van Crevel, Jaap ten Oever, Ilse J. E. Kouijzer

**Affiliations:** 1 Department of Internal Medicine and Radboud Center for Infectious Diseases, Radboud University Medical Center, P.O. Box 9101, 6500 HB Nijmegen, The Netherlands; 2 Department of Medical Imaging, Radboud University Medical Center, Nijmegen, The Netherlands; 3 Department of Internal Medicine, Elisabeth-TweeSteden Hospital, Tilburg, The Netherlands; 4 Department of Medical Microbiology and Radboud Center for Infectious Diseases, Radboud University Medical Center, Nijmegen, The Netherlands; 5 Department of Gastroenterology and Hepatology, Radboud University Medical Center, Nijmegen, The Netherlands

**Keywords:** *Staphylococcus aureus* bacteremia, [^18^F]FDG-PET/CT, Prolonged treatment, Metastatic infection

## Abstract

**Purpose:**

[^18^F]FDG-PET/CT scanning can help detect metastatic infectious foci and reduce mortality in patients with *Staphylococcus aureus* bacteremia (SAB), but it is unknown if patients with SAB and an indication for prolonged treatment because of possible endovascular, orthopaedic implant, or other metastatic infection still need [^18^F]FDG-PET/CT.

**Methods:**

In a retrospective single-center cohort study, we included all consecutive adult patients with SAB between 2013 and 2020 if an [^18^F]FDG-PET/CT scan was performed and antibiotic treatment was planned for ≥ 6 weeks prior to [^18^F]FDG-PET/CT. We aimed to identify patients for whom treatment was adjusted due to the results of [^18^F]FDG-PET/CT, and assessed concordance of [^18^F]FDG-PET/CT and clinical diagnosis for infected prosthetic material.

**Results:**

Among 132 patients included, the original treatment plan was changed after [^18^F]FDG-PET/CT in 22 patients (16.7%), in the majority (*n* = 20) due to diagnosing or rejecting endovascular (graft) infection. Antibiotic treatment modifications were shortening in 2, iv-oral switch in 3, extension in 13, and addition of rifampicin in 4 patients. Ninety additional metastatic foci based on [^18^F]FDG-PET/CT results were found in 69/132 patients (52.3%). [^18^F]FDG-PET/CT suggested vascular graft infection in 7/14 patients who lacked clinical signs of infection, but showed no infection of prosthetic joints or osteosynthesis material in eight patients who lacked clinical signs of such an infection.

**Conclusion:**

[^18^F]FDG-PET/CT can help refine treatment for SAB in patients with clinically suspected endovascular infection or vascular grafts, even if 6 weeks treatment is already indicated, but can be safely omitted in other patients who are clinically stable.

**Supplementary Information:**

The online version contains supplementary material available at 10.1007/s15010-021-01740-4.

## Introduction

*Staphylococcus aureus* bacteremia (SAB) is a serious infection with a mortality of around 20% [1–5]. Metastatic infections, such as abscesses and endovascular infection, are major drivers of mortality. Inadequate treatment and thereby incomplete eradication of bacterial load leads to increased relapses and adverse outcomes [6]. Therefore, by consensus, these patients should receive antibiotics for 4–6 weeks, while antibiotic treatment for uncomplicated bacteremia can safely be shortened to 2 weeks [7].

Earlier studies have shown that up to 71% of metastatic foci are clinically silent [8], leading to an underestimation of the bacterial burden in SAB patients. 2-[^18^F]fluoro-2-deoxy-D-glucose positron emission tomography with combined computed tomography ([^18^F]FDG-PET/CT) has a high sensitivity and specificity for metastatic infectious foci [9, 10]. Integration of [^18^F]FDG-PET/CT into the diagnostic workup of patients with SAB reduces mortality rates [8–10]. Proof of concept studies indicate that [^18^F]FDG-PET/CT can also be used to select patients who are eligible for iv-oral switch [11] or shortening of antibiotic treatment [12]. Therefore, implementation of [^18^F]FDG-PET/CT during the diagnostic work-up might contribute to an individualized treatment plan. We favour a patient-oriented approach to reduce health care expenditure and minimize adverse effects associated with long-term antibiotic treatment. One subgroup of patients in whom [^18^F]FDG-PET/CT might contribute little to SAB management are patients with already diagnosed disseminated infection—and thus an indication for prolonged treatment—before the [^18^F]FDG-PET/CT. It is unknown whether [^18^F]FDG-PET/CT based diagnoses of infectious foci have treatment consequences in this subgroup. In this study, we investigated the impact of [^18^F]FDG-PET/CT in the workup of patients with SAB and an established indication for at least 6 weeks of antibiotic treatment because of possible endovascular, orthopaedic implant, or other metastatic infections.

## Materials and methods

### Study design

In this retrospective cohort study, all consecutive adult patients with SAB between January 2013 and January 2020 at the Radboud university medical center, Nijmegen, the Netherlands, were included if (i) an [^18^F]FDG-PET/CT scan was performed, and (ii) there was an indication for at least 6 weeks of antibiotic treatment prior to this [^18^F]FDG-PET/CT. Exclusion criteria were age < 18 years and death within 72 h after the first positive blood culture. The Regional Institutional Ethics Committee approved this study and the requirement to obtain informed consent was waived (Nr. 2019-6025).

### Outcome measures

The primary outcome of this study was the percentage of patients with an established indication for at least 6 weeks antibiotic treatment whose original treatment plan changed due to the results of [^18^F]FDG-PET/CT. Secondary outcomes were patients with new metastatic foci found by [^18^F]FDG-PET/CT and patient characteristics associated with treatment consequences of [^18^F]FDG-PET/CT. Furthermore, for patients with vascular grafts, prosthetic joints, and/or osteosynthesis material, we assessed the concordance of [^18^F]FDG-PET/CT and clinical assessment for the diagnosis of infected prosthetic material.

### Data collection

The following data were retrieved from the electronic medical charts: demographic characteristics, comorbidity, onset of bacteremia, presence of prosthetic material or intravascular catheters, clinical diagnosis of infectious foci, clinical parameters at the time of the positive blood culture, diagnostic investigations and treatment, antimicrobial therapy, and outcome measures.

### Definitions

SAB was defined as one or more blood cultures positive for *S. aureus* and was considered to be high-risk in case at least one of the following criteria were met: community acquisition, signs of infection for more than 48 h before initiation of appropriate antibiotic treatment, persistent fever more than 72 h after initiation of appropriate antibiotic treatment, persistent positive blood cultures more than 48 h after initiation of appropriate antibiotic treatment, or confirmed metastatic foci at first presentation [1, 13]. Complicated SAB was defined as SAB with deep seated metastatic infections. Comorbidity was reported using the Charlson comorbidity score [14]. A change in original treatment plan was specified into antibiotic treatment modification and/or drainage (surgical and/or radiological). Antibiotic treatment modifications were (i) shortening of antibiotic treatment to 2 weeks, (ii) iv-oral switch after 2 weeks of iv treatment, (iii) extension of iv antibiotic treatment duration, or (iv) addition of rifampicin. Patients were considered to be cured if no signs and symptoms of infection were present 3 months after discontinuation of antibiotic treatment. Relapse of SAB was defined as a second episode of SAB within 3 months after discontinuation of antibiotic treatment.

### Diagnostic workup

In our institution, bedside consultation by an infectious disease (ID) specialist is mandatory in all patients with SAB. In the diagnostic work-up of patients with proven metastatic infection or high-risk SAB both [^18^F]FDG-PET/CT and echocardiography are highly recommended, as described elsewhere [8]. If focal accumulation of [^18^F]FDG was detected, results of the scan were considered abnormal. When the scan results were negative for metastatic infection, they were considered to be true negative if no complicating infectious foci were diagnosed within 2 weeks after the [^18^F]FDG-PET/CT scan was performed. In patients with native valves, transthoracic echocardiography (TTE) was used as a first-line screening for endocarditis. In patients with prosthetic valves and cardiac implantable electronic devices (CIED), transoesophageal echocardiography (TEE) was used as the first line screening. In case of negative TTE for endocarditis, TEE was recommended, especially when imaging quality was limited by anatomical or technical problems. Based on findings at medical history and physical examination, other imaging modalities could be indicated in individual patients.

### Original and final treatment plan

A 2-step approach was used to determine the impact of [^18^F]FDG-PET/CT on original treatment plan. First, the treatment plan before [^18^F]FDG-PET/CT was determined based on clinical information available including medical history, physical examination, and imaging other than [^18^F]FDG-PET/CT. We included possible and proven diagnoses as indicated (Supplemental data, Table S1). Second, the final treatment plan was established based on all clinical information including the primary report of [^18^F]FDG-PET/CT scan and additional imaging indicated by [^18^F]FDG-PET/CT. Interpretation was done by two independent researchers. When consensus was not reached, a third person was consulted to make the final decision. [^18^F]FDG-PET/CT scans were not reviewed. According to the institutional guideline, patients with uncomplicated SAB after performance of echocardiography and [^18^F]FDG-PET/CT, regardless of the presence of risk factors for complicated SAB, are recommended to be treated for 2 weeks [12]. In addition, the institutional guideline recommends iv-oral switch in antimicrobial therapy after 2 weeks of iv treatment in patients with complicated SAB but without evidence of endocarditis after echocardiography and without signs of other endovascular foci on [^18^F]FDG-PET/CT [11].

### Statistical analysis

SPSS (Version 25.0; SPSS, Inc.) was used to analyze the data. Unpaired student’s *t* tests were used to compare normally distributed, continuous variables, otherwise the Mann–Whitney *U* test was used. Categorical variables were compared by use of the chi-square test or Fisher’s exact test when the chi-square test was not appropriate. Differences were considered to be statistically significant at a two-sided *p* value of < 0.05.

## Results

### Patient characteristics

**Fig. 1:**
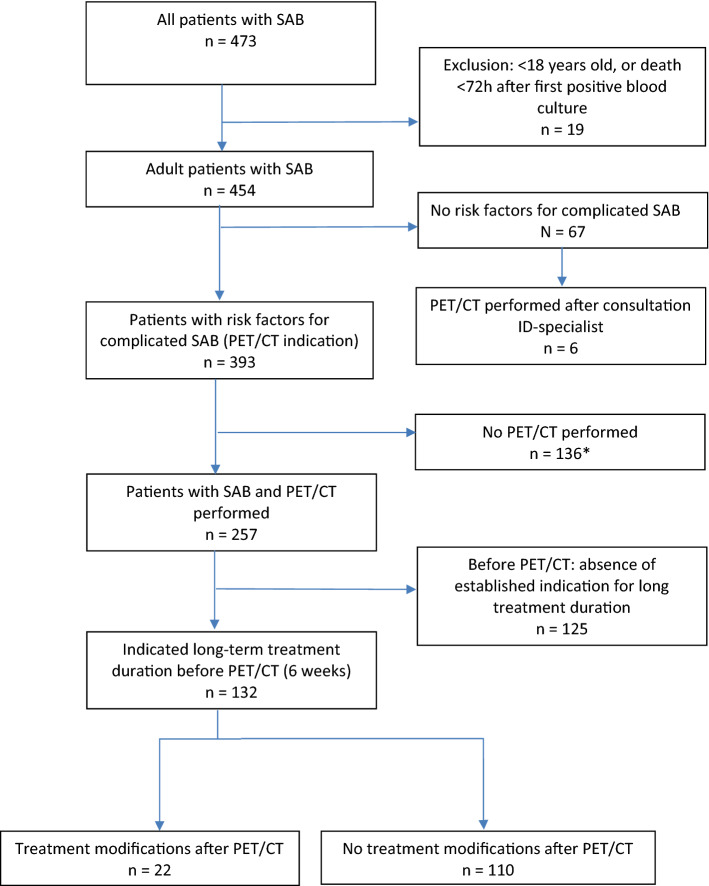
Flowchart of patients included in the study

Between January 2013 and January 2020, 473 patients with SAB were admitted to the Radboudumc. A flowchart of all included patients is shown in Fig. 1. A total of 132 patients with a 6 weeks antibiotic treatment indication before [^18^F]FDG-PET/CT had been performed were included in the analysis. Median time interval between diagnosis of SAB and [^18^F]FDG-PET/CT was 7 days. A low-carbohydrate fat-allowed diet was followed in 80.3% of patients 24 h prior to the scan. For all patients, the mean age was 61 years (range 18–88y), 83 patients were male (62.9%), and 53 patients (40.2%) stayed in the intensive care unit during their hospitalization (Table 1). Two patients (1.5%) had MRSA bacteremia. Three-month mortality was 15.9%.

**Table 1:** Baseline characteristics of all patients

	No treatment modifications after [^18^F]FDG-PET/CT *n* = 110 (%)	Treatment modifications after [^18^F]FDG-PET/CT *n* = 22 (%)	*p* value
Male	68 (61.8)	15 (68.2)	0.57
Age (mean)	61	61	0.81
Community-acquisition Persistent fever > 72 h Persistent positive blood culture > 48 h Delay between onset and start treatment > 48 h Prosthetic material present	58 (52.7) 48 (43.6) 52 (47.3) 61 (55.5) 62 (56.4)	11 (50.0) 12 (54.5) 13 (59.1) 11 (50.0) 8 (36.4)	0.82 0.41 0.27 0.61 0.09
ID specialist bed side consultation	102 (92.7)	22 (100.0)	0.19
Charlson comorbidity score Diabetes Mellitus Malignancy Renal failure Immunocompromised Joint prosthesis Osteosynthesis Heart valve prosthesis Vascular graft prosthesis Pacemaker/ICD in situ	3 21 (19.1) 14 (12.7) 9 (8.2) 20 (18.2) 20 (18.2) 17 (15.5) 19 (17.3) 16 (14.5) 7 (6.4)	3 5 (22.7) 5 (22.7) 0 (0.0) 4 (18.2) 2 (9.1) 0 (0.0) 3 (13.6) 6 (27.3) 2 (9.1)	0.46 0.69 0.22 0.17 1.00 0.30 0.05 0.68 0.14 0.64
3-month outcome			
Cured	90 (81.8)	16 (72.7)	0.20
Relapse	1 (0.9)	1 (4.5)	0.21
Mortality	16 (14.5)	5 (22.7)	0.37

### Detection of infectious foci using [^18^F]FDG-PET/CT

**Fig. 2:**
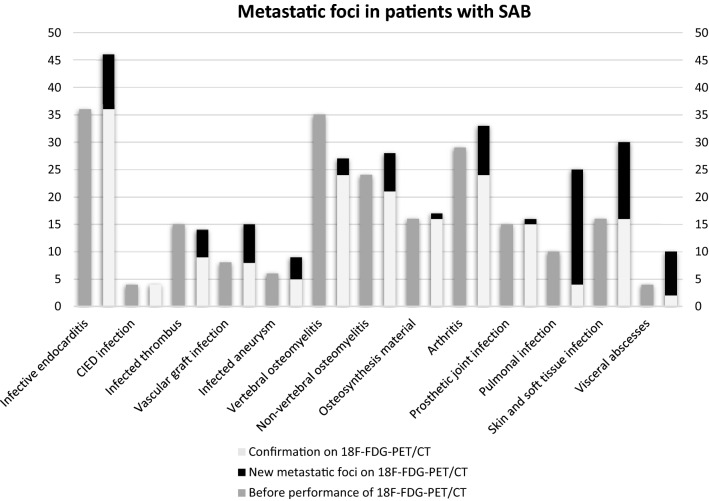
Prevalence of metastatic foci before and after the performance of [^18^F]FDG-PET/CT

Ninety additional metastatic foci based on [^18^F]FDG-PET/CT results were found in 69 in 132 patients (52.3%) (Fig. 2). In all 132 patients, [^18^F]FDG-PET/CT confirmed 184 diagnoses of infectious foci and rejected 34 possible diagnoses based on clinical suspicion. In 87 of 132 patients (65.9%), a total of 129 conventional imaging procedures were performed before [^18^F]FDG-PET/CT: 49 non-cardiac ultrasounds, 52 CT scans, and 28 MR scans.

### Changes in the original treatment plan based on [^18^F]FDG-PET/CT

In the overall study cohort, in 22 out of 132 patients (16.7%), indicated treatment was changed after [^18^F]FDG-PET/CT. In the remaining 110 patients (83.3%), [^18^F]FDG-PET/CT did not influence the original treatment plan. In the 22 patients with a total of 27 treatment modifications prompted by [^18^F]FDG-PET/CT, 14 patients had one modification in antibiotic treatment, four patients had two antibiotic modifications (including one patient with additional interventions), and four underwent extra interventions only. With respect to antibiotic treatment modifications, treatment duration was shortened to 2 weeks in two patients (9.1%), iv-oral switch was performed in three (13.6%), iv antibiotic treatment duration was extended in nine patients (40.9%), and in four patients both iv antibiotic treatment was extended and rifampicin was added (18.2%) (Table 2). All five patients with additional drainage based on [^18^F]FDG-PET/CT results had persistent fever (five patients) or persistent positive blood cultures (two patients). Drainage in these five patients was performed radiologically in two patients: one patient with septic arthritis and one patient with infected ascites. Surgical drainage was performed in three patients: vascular graft with aortic valve replacement in one patient, debridement of an infected aneurysm in another patient, and surgical debridement of an infected posttraumatic hematoma in the third patient.

**Table 2:** Antibiotic treatment modifications (22) based on [^18^F]FDG-PET/CT results in 18 patients

Treatment modification	Patients	Diagnosis before [^18^F]FDG-PET/CT scan	Diagnosis after [^18^F]FDG-PET/CT scan
Shortening treatment^a^	No. 1	Infected thrombus	No metastatic infection
	No. 2	Arthritis	No metastatic infection
Iv oral switch^b^	No. 3	Infected thrombus	No infected thrombus; proven pulmonary metastatic infection
	No. 4	Infected thrombus	No infected thrombus; proven pulmonary metastatic infection
	No. 5	Infected thrombus	No infected thrombus; proven pulmonary metastatic infection
Extension of iv treatment^c^	No. 6	Non-vertebral osteomyelitis	Endocarditis, epidural abscesses, non-vertebral osteomyelitis
	No. 7	Non-vertebral osteomyelitis	Endocarditis and non-vertebral osteomyelitis
	No. 8	Liver abscess	Endocarditis and liver abscess
	No. 9	Soft tissue abscess	Infected thrombus and soft tissue abscess
	No. 10	Arthritis	Infected aneurysm, arthritis, abscesses
	No. 11	Vertebral osteomyelitis and arthritis	Epidural abscesses and arthritis
	No. 12	Prosthetic joint infection	Endocarditis and prosthetic joint infection
	No. 13	Endocarditis prosthetic valve	Endocarditis and vascular graft infection
	No. 14	Endocarditis prosthetic valve	Endocarditis and vascular graft infection
Extension of iv treatment^c^ and addition of rifampicin	No. 15	Vertebral osteomyelitis	Vertebral osteomyelitis, infected aneurysm
	No. 16	Endocarditis native valve, cardiac implantable device infection	Endocarditis, cardiac implantable device infection, infected thrombus and vascular graft
	No. 17	Endocarditis native valve	Endocarditis and vascular graft infection
	No. 18	Endocarditis native valve	Endocarditis, infected aneurysm and vascular graft infection

^a^Shortening of treatment: 2 weeks iv

^b^iv-oral switch: 2 weeks iv and ≥ 4 weeks orally

^c^Extension of iv treatment: iv-oral switch to 6 weeks iv or ≥ 6 weeks iv

### Impact of [^18^F]FDG-PET/CT in SAB patients with prosthetic material

The type of prosthetic material was associated with the additional value of [^18^F]FDG-PET/CT. Seventy of the 132 patients had together 92 prostheses (Table 1). Of the 22 patients with vascular prostheses, eight patients were considered to have a vascular graft infection based on CT or during surgery. [^18^F]FDG-PET/CT confirmed the diagnosis of vascular graft infection in all these eight patients. In addition, [^18^F]FDG-PET/CT showed vascular graft infection in eight of the remaining 14 patients (43%) without clinical suspicion, of whom one patient had probable infection and five patients had proven infection according to the MAGIC criteria [15]. These [^18^F]FDG-PET/CT results led to extension of iv treatment and/or addition of rifampicin in five patients.

Of all 22 patients with prosthetic joints, [^18^F]FDG-PET/CT confirmed all 15 clinically suspected prosthetic joint infections. Of seven patients without clinical suspicion of prosthetic joint infection, one showed [^18^F]FDG uptake on [^18^F]FDG-PET/CT. This patient was treated for 3 months with antibiotics because of vertebral osteomyelitis with paravertebral abscesses (diagnosis made by MRI). The increased [^18^F]FDG uptake around total hip and knee prostheses was considered to be related to inflammation due to particle wear rather than infection. No intervention was performed. No relapse occurred during the 3 months follow-up after discontinuation of antibiotic treatment.

Of all 17 patients with osteosynthesis material, [^18^F]FDG-PET/CT confirmed all 16 clinically suspected infections. In the single patient with clinically unsuspected osteosynthesis material [^18^F]FDG-PET/CT showed no increased uptake.

### Impact of [^18^F]FDG-PET/CT in SAB patients without prosthetic material

Of 132 patients, 62 patients (47.0%) had no prosthetic material in situ. Of these 62 patients, 14 patients (22.6%) had a change in treatment plan: three additional drainage (4.8%) and 11 antibiotic treatment modifications (17.7%). The 11 antibiotic treatment modifications in these patients were due to iv-oral switch (3 patients), shortening of treatment (2 patients), and extension of iv antibiotic treatment (6 patients) (Table 2). Of these 11 antibiotic treatment modifications, 5 were due to detection and 4 were due to rejection of an endovascular infection (Table 2). In 3 out of 34 patients (25.8%) with native valve endocarditis this diagnosis was based on increased [^18^F]FDG uptake of the heart valve region, leading to prolonged iv treatment instead of the original treatment plan of iv-oral switch. Signs of endocarditis were absent on TEE in 2 of these 3 patients. Of these 3 patients, 2 patients had persistent fever despite adequate antibiotic treatment. In the other patient without persistent fever or persistent positive blood cultures, epidural abscesses were found on MRI after [^18^F]FDG-PET/CT showed vertebral osteomyelitis, leading to 6 weeks of iv antibiotic treatment instead of oral stepdown.

## Discussion

This study showed that in only 16.7% of SAB patients with indicated antibiotic treatment for ≥ 6 weeks prior to [^18^F]FDG-PET/CT because of known metastatic infection, [^18^F]FDG-PET/CT changed the original treatment plan, including both treatment intensification and de-intensification. These modifications were almost exclusively due to the detection or rejection of endovascular infections (Table 2). Our study confirmed the high sensitivity of [^18^F]FDG-PET/CT for detection of additional infectious foci: 90 new metastatic infections in 69 of 132 patients (52.3%) were diagnosed. However, only few had treatment consequences. All 5 patients with additional drainage after [^18^F]FDG-PET/CT had persistent fever and/or persistent positive blood cultures and therefore suspicion for a complicated course.

Our data confirmed that the diagnosis of vascular graft infection can be easily missed on conventional imaging, because symptoms and signs are often absent or non-specific [15]. For this reason, [^18^F]FDG-PET/CT has become an important part of the work-up of vascular graft infection [15], of course in addition to all clinical parameters as increased [^18^F]FDG uptake can also be found in noninfectious vascular prostheses [16]. For the diagnosis of prosthetic valve endocarditis and CIED infection, the value of [^18^F]FDG-PET/CT has already been determined and has been incorporated in international guidelines [17, 18]. For prosthetic joints and osteosynthesis material, clinical examination proved to be excellent for establishing an infection or rejecting it, obviating the need for [^18^F]FDG-PET/CT in these patients [19, 20]. In patients without prosthetic material, 17.7% of patients had antibiotic treatment modification and all were a direct consequence of our institutional policy to reduce treatment duration or to switch to oral treatment in patients without metastatic infections or without endovascular infections, respectively. Therefore, the findings of our study have implications for three groups of patients who already have an indication for long-term treatment before an [^18^F]FDG-PET/CT scan has been performed. First, patients with a vascular prosthesis, in whom the diagnosis of a vascular graft infection may lead to continuation of iv treatment and the addition of rifampicin. Second, the patients without vascular prosthetic material and a high suspicion of an endovascular infection, such as those with venous thrombosis or prolonged bacteremia. In these patients, [^18^F]FDG-PET/CT allows for oral stepdown if endovascular infection including endocarditis has been ruled out [11]. Finally, in patients with deterioration and/or persistent fever or persistent positive blood cultures that is clinically unexplained, [^18^F]FDG-PET/CT should be considered to search for complications of SAB.

The results of this study contribute to the individualized diagnostic work-up and therapeutic management of patients with high-risk SAB, in whom on group-level [^18^F]FDG-PET/CT has been shown to reduce mortality [8, 9]. Additionally, [^18^F]FDG-PET/CT—in combination with echocardiography—has additional value in selecting patients in whom treatment can be safely shortened to 2 weeks of treatment [12] or oral step down [11]. In general, we advocate to make an [^18^F]FDG-PET/CT early in the course of SAB. Due to the high sensitivity of this modality for infectious foci, this strategy could provide a ‘one-stop-shop’ to optimally stage patients with SAB, potentially avoiding delayed diagnoses, multiple imaging procedures, and associated cost. One exception is the patient with vertebral osteomyelitis and neurologic symptoms in whom MRI should be performed, as the detection of epidural abscesses by [^18^F]FDG-PET/CT is limited [21] and may have important treatment consequences. The current study is the most detailed study to date on the additional value of [^18^F]FDG-PET/CT in patients with complicated SAB and an established long-term antibiotic treatment indication and provides valuable observations on the optimal use of [^18^F]FDG-PET/CT in these patients. Two other studies on treatment modification after [^18^F]FDG-PET/CT analyzed different patient populations including other type of micro-organisms and also uncomplicated SAB. Therefore, comparing these studies is difficult. One study with both SAB patients with and without long-term antibiotic treatment indication, showed that [^18^F]FDG-PET/CT led to 104 treatment modifications in 74 patients [8]. In another study including 40 patients with infective endocarditis, only 8 due to *S. aureus,* [^18^F]FDG-PET/CT findings changed treatment plan in 14 patients (35%) [22].

Within the limitations of a retrospective design and without the possibility of reviewing all scans, our study is the largest so far on investigating the role of [^18^F]FDG-PET/CT on treatment modifications in SAB, using objective criteria for diagnosis of metastatic infection in SAB (Supplemental data, Table S1). Our primary endpoint was influenced by two factors. Nuclear imaging was omitted in 136 patients with risk factors for a complicated SAB, some of whom might have fulfilled our clinical inclusion criteria. This mainly concerned patients with less comorbidity and fewer risk factors for a complicated course (Supplementary data, Table S2). This selection bias probably has led to an overestimation of the value of [^18^F]FDG-PET/CT. Also, the total number of conventional imaging performed prior to the [^18^F]FDG-PET/CT likely had a negative influence on the additional value of [^18^F]FDG-PET/CT in diagnosing extra metastatic foci. Finally, for stronger conclusions on omitting [^18^F]FDG-PET/CT in specific patient groups, larger prospective studies, including health technology assessments, are needed. Based on these results, a personalized diagnostic work-up and management may become within reach for each individual patient.

In conclusion, [^18^F]FDG-PET/CT in the diagnostic work-up for patients with complicated SAB and an indicated treatment duration of at least 6 weeks has most impact for patients with suspicion on endovascular infection including vascular graft infection. For other clinically stable patients with complicated SAB and long-term indicated treatment, including patients with prosthetic joints and osteosynthesis material without clinical signs of infection of this prosthetic material, our findings suggest that [^18^F]FDG-PET/CT can be safely omitted during initial diagnostic workup.

## Supplementary Information

Below is the link to the electronic supplementary material.

Supplementary file1 (DOCX 20 KB)

## Data Availability

To be requested from the authors.
