# Supplementary material for: Individualizing the use of [18F]FDG-PET/CT in patients with complicated Staphylococcus aureus bacteremia: experiences from a tertiary care center
Source: Infection. 2021 Dec 20;50(2):491–8. doi: 10.1007/s15010-021-01740-4 (PMC8942890; doi:10.1007/s15010-021-01740-4)
Supplement: Supplementary file 1 — Supplementary file1 (DOCX 20 KB) [file 15010_2021_1740_MOESM1_ESM.docx]

**Supplementary data
Table S1. Diagnostic criteria for metastatic infection in patients with SAB and indicated treatment**

|  | **Diagnostic work-up**  **excluding [^18^F]FDG-PET/CT** | **Diagnostic work-up including [^18^F]FDG-PET/CT** | **Indicated treatment** |
| --- | --- | --- | --- |
| Infective endocarditis with or without prosthetic valve | Possible: ESC 2015 modified diagnostic criteria [17]  Proven: ESC 2015 modified diagnostic criteria [17] | Possible: ESC 2015 modified diagnostic criteria [17]  Proven: ESC 2015 modified diagnostic criteria [17] | 6 wks iv + valve replacement if indicated |
| Cardiac implantable electronic device | Local device infection: clinical signs of pocket infection  Possible and proven CIED lead infection: ESC 2015 modified diagnostic criteria [17] and 2020 International CIED Infection Criteria [18] | Local device infection: 2020 International CIED Infection Criteria [18]  Possible and proven CIED lead infection: ESC 2015 modified diagnostic criteria [17] and 2020 International CIED Infection Criteria [18] | Surgical removal of the CIED (preferably) + 2-6 wks iv (dependent on blood culture result and valve vegetations after CIED extraction) |
| Infected thrombus | Possible: radiological (US or CT) signs of thrombus  Proven: CT proven infected thrombus [23] | Proven: suspicious metabolic activity on [^18^F]FDG-PET/CT [24] | 6 wks iv |
| Infected aneurysm | Possible: radiological (US or CT) signs of aneurysm  Proven: CTa-proven infected aneurysm [25] or infected aneurysm found during surgery | Proven: suspicious metabolic activity on [^18^F]FDG-PET/CT [25] | 6 wks iv |
| Vascular graft infection | Possible: minor radiological signs of vascular graft infection [15]  Proven: major radiological signs of vascular graft infection [15] or infection found during surgery | Proven: suspicious metabolic activity on [^18^F]FDG-PET/CT [15,26] | Surgical drainage (if possible) + >6 wks iv + rifampicin |
| Prosthetic joint infection | Possible: new onset pain of joint prosthesis  Proven: new onset clinical or radiological signs of septic arthritis | Proven: suspicious metabolic activity on [^18^F]FDG-PET/CT [27] | Surgical drainage + 2 wks iv + >4wks po (total duration 12 wks) + rifampicin |
| Infected osteosyntheses material | Possible: new onset pain of osteosynthesis material  Proven: new onset clinical or radiological signs of infected osteosynthesis material | Proven: suspicious metabolic activity on [^18^F]FDG-PET/CT [27] | Surgical drainage + 2 wks iv + >4wks po (total duration 12 wks) + rifampicin |
| (Non-) vertebral osteomyelitis | Possible: new onset pain of vertebrae or bone  Proven: MRI-proven osteomyelitis [28] | Proven: suspicious metabolic activity on [^18^F]FDG-PET/CT [28] | 2 wks iv + 4 wks po  Epidural abscess: surgical drainage (in case of neurological signs) + 6 wks iv |
| Septic arthritis | Possible: new onset joint pain  Proven: new onset clinical or radiological signs of arthritis | Proven: suspicious metabolic activity on [^18^F]FDG-PET/CT [29] | Drainage + 2 wks iv + 4 wks po |
| Visceral abscess | Possible: encapsulated fluid collection  Proven: Radiologically diagnosed visceral abscess | Proven: abscess configuration with metabolic activity on [^18^F]FDG-PET/CT [29] | Radiological drainage + 2 wks iv + 4 wks po |
| Pulmonary foci | Possible: bilateral consolidations on X-ray  Proven: bilateral consolidations on CT | Proven: suspicious metabolic activity on [^18^F]FDG-PET/CT [30] | 2 wks iv + 4 wks po |

|  | **No performance of [^18^F]FDG-PET/CT n = 112^a^ (%)** | **Performance of [^18^F]FDG-PET/CT n = 132 (%)** | ***p* value** |
| --- | --- | --- | --- |
| Male | 66 (58.9) | 83 (62.9) | 0.53 |
| Age (mean) | 58 | 61 | 0.16 |
| Community-acquisition  Persistent fever >72h  Persistent positive blood culture >48h  Delay between onset and start treatment >48h  Prosthetic material present | 17 (15.2)  36 (32.1)  20 (17.9)  27 (24.1)  38 (33.9) | 69 (52.3)  60 (45.5)  65 (49.2)  72 (54.5)  70 (53.0) | <0.01  0.04  <0.01  <0.01  0.01 |
| ID specialist bed side consultation | 84 (75.0) | 124 (93.9) | <0.01 |
| Charlson comorbidity score (median)  -Diabetes Mellitus  -Malignancy  -Renal failure  -Immunocompromised  -Joint prosthesis  -Osteosynthesis  -Heart valve prosthesis  -Vascular graft prosthesis  -Pacemaker/ICD in situ | 0  20 (17.9)  31 (27.7)  5 (4.5)  30 (26.8)  12 (10.7)  10 (8.9)  6 (5.4)  8 (7.1)  8 (7.1) | 3  26 (19.7)  19 (14.4)  9 (6.8)  24 (18.2)  22 (16.7)  17 (12.9)  22 (16.7)  22 (16.7)  9 (6.8) | 0.71  0.01  0.43  0.14  0.18  0.33  0.01  0.02  0.92 |

In 136 patients with at least one risk factor for metastatic infection, no [^18^F]FDG-PET/CT was performed. Reasons for not performing an [^18^F]FDG-PET/CT were expected lack of consequences for treatment (99 patients, 72.8%), choice for palliative policy (24 patients, 17.6%), or early death during treatment (13 patients, 9.6%). Characteristics of patients with and without performance of [^18^F]FDG-PET/CT, excluding patients with death within 72h after first positive blood cultures and abstinent policy, are shown in Table 4. Compared to the 132 patients with performance of [^18^F]FDG-PET/CT, patients without [^18^F]FDG-PET/CT had less comorbidity and less risk factors for complicated SAB.

**Table S2. Characteristics in high-risk SAB patients with and without performance of [^18^F]FDG-PET/CT**

^a^ excluding the 24 patients with abstinent policy after first positive blood cultures using the same
exclusion criteria as the study group of 132 patients.

**Additional References**

23. Mori H, Fukuda T, Isomoto I, et al. CT diagnosis of catheter-induced septic thrombus of vena cava. *J Comput Assist Tomogr*. 1990;14(2):236-8.

24. Bleeker-Rovers CP, Jager G, Tack CJ, et al. F-18-fluorodeoxyglucose positron emission tomography leading to a diagnosis of septic thrombophlebitis of the portal vein: description of a case history and review of the literature. *J Intern Med*. 2004;255(3):419-23.

25. Wilson WR, Bower TC, Creager MA, et al. Vascular Graft Infections, Mycotic Aneurysms, and Endovascular Infections: A Scientific Statement From the American Heart Association. *Circulation*. 2016;134(20):e412-e60.

26. Chakfe N, Diener H, Lejay A, et al. Editor's Choice - European Society for Vascular Surgery (ESVS) 2020 Clinical Practice Guidelines on the Management of Vascular Graft and Endograft Infections. *Eur J Vasc Endovasc Surg*. 2020;59(3):339-84.

27. Osmon DR, Berbari EF, Berendt AR, et al. Diagnosis and management of prosthetic joint infection: clinical practice guidelines by the Infectious Diseases Society of America. *Clin Infect Dis*. 2013;56(1):e1-e25.

28. Berbari EF, Kanj SS, Kowalski TJ, et al. 2015 Infectious Diseases Society of America (IDSA) Clinical Practice Guidelines for the Diagnosis and Treatment of Native Vertebral Osteomyelitis in Adults. *Clin Infect Dis*. 2015;61(6):e26-46.

29. Jamar F, Buscombe J, Chiti A, et al. EANM/SNMMI guideline for 18F-FDG use in inflammation and infection. *J Nucl Med*. 2013;54(4):647-58.

30. Mendez-Echevarria A, Coronado-Poggio M, Baquero-Artigao F, et al. Septic pulmonary emboli detected by (18)F-FDG PET/CT in children with *S. aureus* catheter-related bacteremia. *Infection*. 2017;45(5):691-6.
